# Non-linear association of anthropometric measurements and pulmonary function

**DOI:** 10.1038/s41598-021-93985-0

**Published:** 2021-07-16

**Authors:** Rui-Heng Zhang, Jian-Bo Zhou, Yao-Hua Cai, Lin-Ping Shu, Jinkui Yang, Wenbin Wei, Albert Lecube

**Affiliations:** 1 Beijing Tongren Hospital, Capital Medical University, Beijing, China; 2 Department of Endocrinology, Beijing Tongren Hospital, Capital Medical University, Beijing, China; 3 Department of Opthalmology, Beijing Tongren Hosptial, Capital Medical University, Beijing, China; 4 Obesity, Diabetes and Metabolism Research Group (ODIM), Endocrinology and Nutrition Department, Institut de Recerca Biomèdica de Lleida (IRBLleida), Hospital Universitari Arnau de Vilanova, Universitat de Lleida, Lleida, Catalonia Spain

**Keywords:** Endocrine system and metabolic diseases, Public health, Weight management

## Abstract

This study examined the association of anthropometric measurements [body mass index (BMI), waist circumference (WC), percentage body fat (PBF), body roundness index (BRI) and A Body Shape Index (ABSI)] with pulmonary function using a United States national cohort. This cross-sectional study included 7346 participants. The association between anthropometric measurements and pulmonary function was assessed by multivariable linear regression. Where there was evidence of non-linearity, we applied a restricted cubic spline to explore the non-linear association. All analyses were weighted to represent the U.S. population and to account for the intricate survey design. After adjusting for age, race, education, smoking, and physical activity, both underweight and obesity were associated with reduced forced expiratory volume in 1 s (FEV_1_) and forced vital capacity (FVC). Furthermore, the associations between BMI and FEV_1,_ as well as FVC, were reversed U-shape in both males and females. Similar non-linear association shape occurred in WC, PBF, BRI and ABSI. Conclusion: BMI, WC, PBF, BRI, ABSI are non-linearly associated with pulmonary function. Reduced pulmonary function is a risk factor for future all-cause mortality and cardiovascular events; thus, this nonlinearity may explain the U-shape or J-shape association of BMI with overall mortality and cardiovascular events.

## Introduction

Over the past ~ 50 years, the prevalence of obesity has increased worldwide, ranging from 3.7% in Japan to 38.2% in the United States when using body mass index ≥ 30 kg/m^2^ as cut-off value^1^. In addition, obesity has become a major public health concern, with enormous physical, social, economic, and psychological consequences^2,3^. For example, obesity increases the risk of many serious diseases and health conditions, such as type 2 diabetes, cardiovascular diseases, obstructive sleep apnea, non-alcoholic fatty liver disease, and cancer^4,5^. Furthermore, cross-sectional studies have also reported a reduction in forced expiratory volume in 1 s (FEV_1_) and forced vital capacity (FVC) in obese participants, suggesting that excess body weight has deleterious effects on pulmonary function either directly or indirectly^6–9^.

However, results showing the negative impact of excess body weight are based mainly on the unverified assumption that the excess body weight is negatively and linearly associated with pulmonary function. For example, in a large cross-sectional study of the Korean population, being underweight was also associated with an impaired pulmonary function^10^. This data suggests a complex association between lung function and anthropometric measures. Additionally, whether abdominal obesity or total body fat has a disparate association with lung function compared to traditional anthropometric measure (BMI) has not been previously assessed in large populations.

Therefore, this study sought to examine in detail the association between anthropometric measures [body mass index (BMI), waist circumference (WC), percentage body fat (PBF), body roundness index (BRI), and A body shape index (ABSI)] and pulmonary function using data from the 2007–2012 National Health and Nutrition Examination Survey (NHANES), a U.S. national cohort.

## Results

### Cross-sectional characteristics of the participants

**Figure 1:**
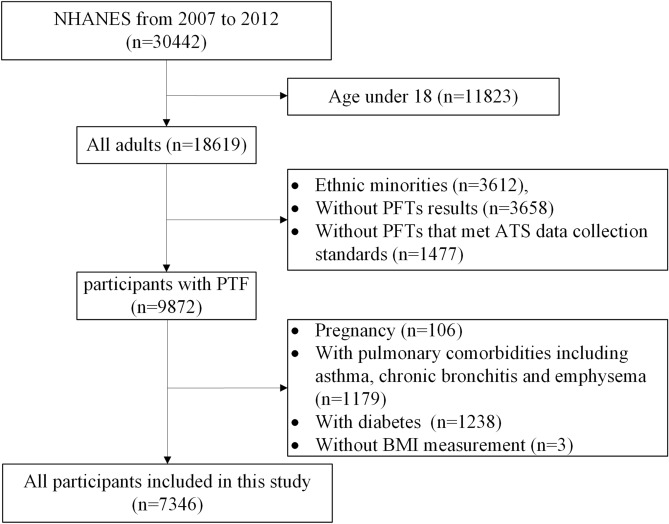
Flow gram of included participants. *PFTs* pulmonary function tests, *ATS* American Thoracic Society.

The primary clinical and pulmonary data of the 7346 participants (Fig. 1) that were included in this cross-sectional study according to their BMI classification (Table 1). The weighted proportions of underweight, normal weight, overweight, and obesity were 1.5%, 32.0%, 35.4%, and 31.0%, respectively. Compared to participants with normal weight, those with obesity were significantly older and more likely to be male, Mexican American, and non-Hispanic Black, with lower educational level, physically inactive, and non-smokers. We first examined the correlation among anthropometric measurements (Supplementary Table 1 and Supplementary Fig. 1). All anthropometric measurements were closely correlated with each other (R-squared value ranged from 0.761 to 0.917), except for ABSI. ABSI showed good allometric characteristics with other measurements. Both underweight and obesity groups exhibited significantly reduced FEV_1_ and FVC measurements in comparison to normal-weight participants, without significant differences between participants with normal weight and overweight. The association remained significant after adjusting for age, race, educational level, physical activity (MET score), and smoking status in both males and females (Table 2). Additionally, obesity was associated with a slightly elevated FEV_1_/FVC ratio, indicating a restrictive pulmonary dysfunction pattern.

**Table 1:** Cross-sectional characteristics of participants.

	Underweight (n = 108)	Normal weight (n = 2198)	Overweight (n = 2557)	Obesity (n = 2483)
Weighted proportion, %(n = 7346)	1.5 (0.2)	32.0 (1.0)	35.4 (0.9)	31.0 (0.8)
Age (years) (n = 7346)	36.9 (1.8)*	40.4 (0.7)	44.9 (0.5)*	44.0 (0.5) *
Male %(n = 7346)	21.5 (4.6)*	43.2 (1.3)	57.5 (1.2)*	49.0 (1.3) *
**Ethnicity % (n = 7346)**				
Mexican American	4.2 (1.8)	7.0 (0.9)	11.2 (1.5) *	11.4 (1.6) *
Non-Hispanic White	85.1 (2.8)	83.7 (1.4)	79.6 (1.9) *	73.8 (2.6) *
Non-Hispanic Black	10.8 (1.9)	9.3 (1.0)	9.2 (1.0)	14.8 (1.8) *
Education level (%)(n = 7331)	26.7 (5.5)	34.6 (1.9)	32.3 (1.9)	24.9 (1.5) *
Current smoker % (n = 7346)	28.0 (5.2)	21.5 (1.6)	16.7 (1.2) *	13.8 (0.9) *
MET scores/week (n = 7346)	480 [120–1080]	600 [180–1320]	540 [160–1440]	400 [80–1320]
HOMA-IR (n = 3038)	0.9 [0.6–1.2]*	1.4 [1.0–2.0]	2.3 [1.6–3.4] *	3.6 [2.5–5.3] *
C-reactive protein (mg/dL) (n = 5165)	0.03 [0.01–0.07] *	0.07 [0.03–0.18]	0.15 [0.07–0.30] *	0.31 [0.15–0.66] *
BMI (kg/m^2^) (n = 7346)	17.75 (0.07) *	22.42 (0.06)	27.36 (0.04) *	35.26 (0.16) *
Height (cm) (n = 7346)	166.8 (0.7) *	169.8 (0.2)	170.7 (0.3) *	169.7 (0.3)
Waist circumference (cm) (n = 7206)	70.9 (0.5) *	82.5 (0.3)	95.8 (0.2) *	112.6 (0.4) *
Percentage body fat (%) (n = 4598)	24.3 (0.5) *	29.2 (0.2)	33.2 (0.2) *	39.0 (0.3) *
Body roundness index (n = 7038)	2.09 (0.05) *	3.13 (0.03)	4.62 (0.02) *	7.06 (0.05) *
A body shape index (n = 7206)	0.0808 (0.0006) *	0.0797 (0.0002)	0.0808 (0.0001) *	0.0807 (0.0001) *
FEV_1_ (%predicted) (n = 7346)	90.8 (1.8) *	98.0 (0.5)	98.6 (0.3)	96.5 (0.4) *
FVC (%predicted) (n = 7346)	92.9 (1.4) *	101.7 (0.4)	101.5 (0.3)	98.3 (0.4) *
FEV_1_/FVC ratio (n = 7346)	80.6 (1.1) *	79.5 (0.4)	77.8 (0.2) *	79.0 (0.2)

Data were weighted estimates and expressed as mean (standard error) or median [percentile 25–percentile 75] (n = number of participants in analysis). Education level, percentage of participants, completed college graduate or above.

*HOMA-IR* homeostasis model of assessment for insulin resistance index, *MET score* metabolic equivalent scores per week.

*p < 0.05 compared to normal weight.

**Table 2:** Association of body weight and pulmonary function.

		Underweight (n = 108)	Normal weight (n = 2198)	Overweight (n = 2557)	Obesity (n = 2483)
Female	FEV_1_ %predicted	**− 6.58 [−10.95, −2.21]**	Reference	0.30 [−0.86, 1.47]	**−1.35 [−2.65, −0.06]**
	FVC %predicted	**−8.48 [−11.90, −5.06]**	Reference	−0.03 [−1.16, 1.09]	**−2.92 [−4.14, −1.70]**
	FEV_1_/FVC ratio %	1.08 [−0.62, 2.77]	Reference	0.15 [−0.46, 0.75]	**1.03 [0.41, 1.65]**
Male	FEV_1_ %predicted	**−9.43 [−14.28, −4.57]**	Reference	1.16 [−0.29, 2.62]	**−1.58 [−2.92, −0.23]**
	FVC %predicted	**−9.82 [−14.86, −4.77]**	Reference	0.05 [−1.29, 1.38]	**−3.23 [−4.57, −1.89]**
	FEV_1_/FVC ratio %	0.52 [−3.58, 4.62]	Reference	0.84 [−0.01, 1.68]	**1.19 [0.45, 1.92]**

Data were weighted estimates and expressed as mean [95% confidence interval].

Multiple linear regression was adjusted for age, race, education level, physical activity (MET score), smoking status.

Bold: p < 0.05.

The association between obesity and pulmonary function no longer existed after additional adjustment for insulin resistance or C-reactive protein (CRP, Supplementary Table 2). We then determined the relationship between obesity (BMI ≥ 30) and pulmonary function, after stratifying by insulin resistance or CRP. Obesity was associated with lower FEV_1_, FVC, and higher FEV_1_/FVC ratio only in the highest insulin resistance/CRP tertile (Supplementary Table 3). We performed a sensitivity analysis by including only non-smokers. The sensitivity analysis excluded the possibility of residual confounding by smoking (Supplementary Table 4).

### Reversed U-shape association between BMI, WC, PBF, BRI, ABSI and pulmonary function

**Figure 2:**
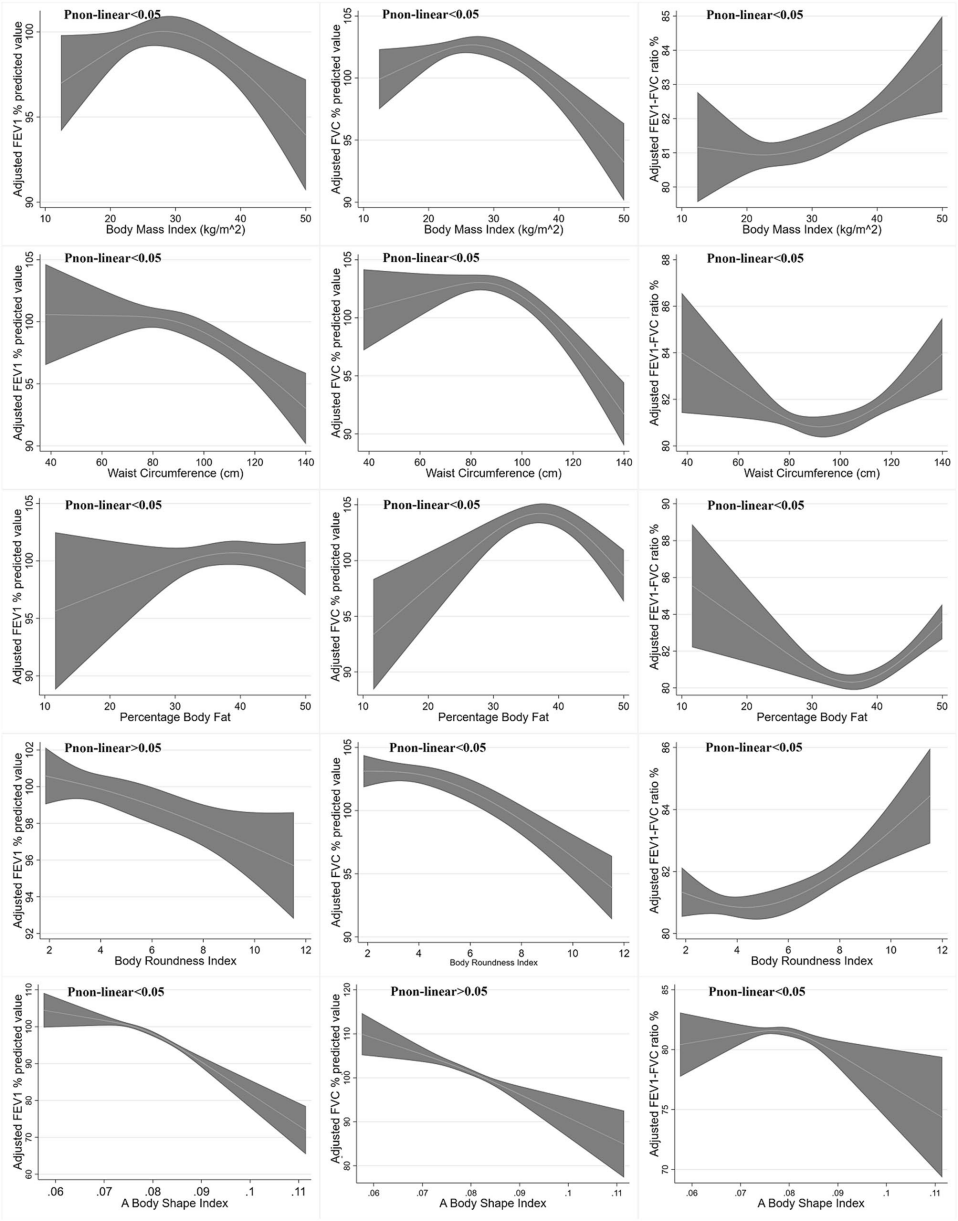
Reverse U-shaped association between BMI, WC, PBF, BRI, ABSI and pulmonary function in female. Data were weighted estimates. The shadow area represents a 95% confidence interval. Pi_non-linear_ was estimated by a two-line piecewise linear model. The model was adjusted for age, race, education, smoking, physical activity.

**Figure 3:**
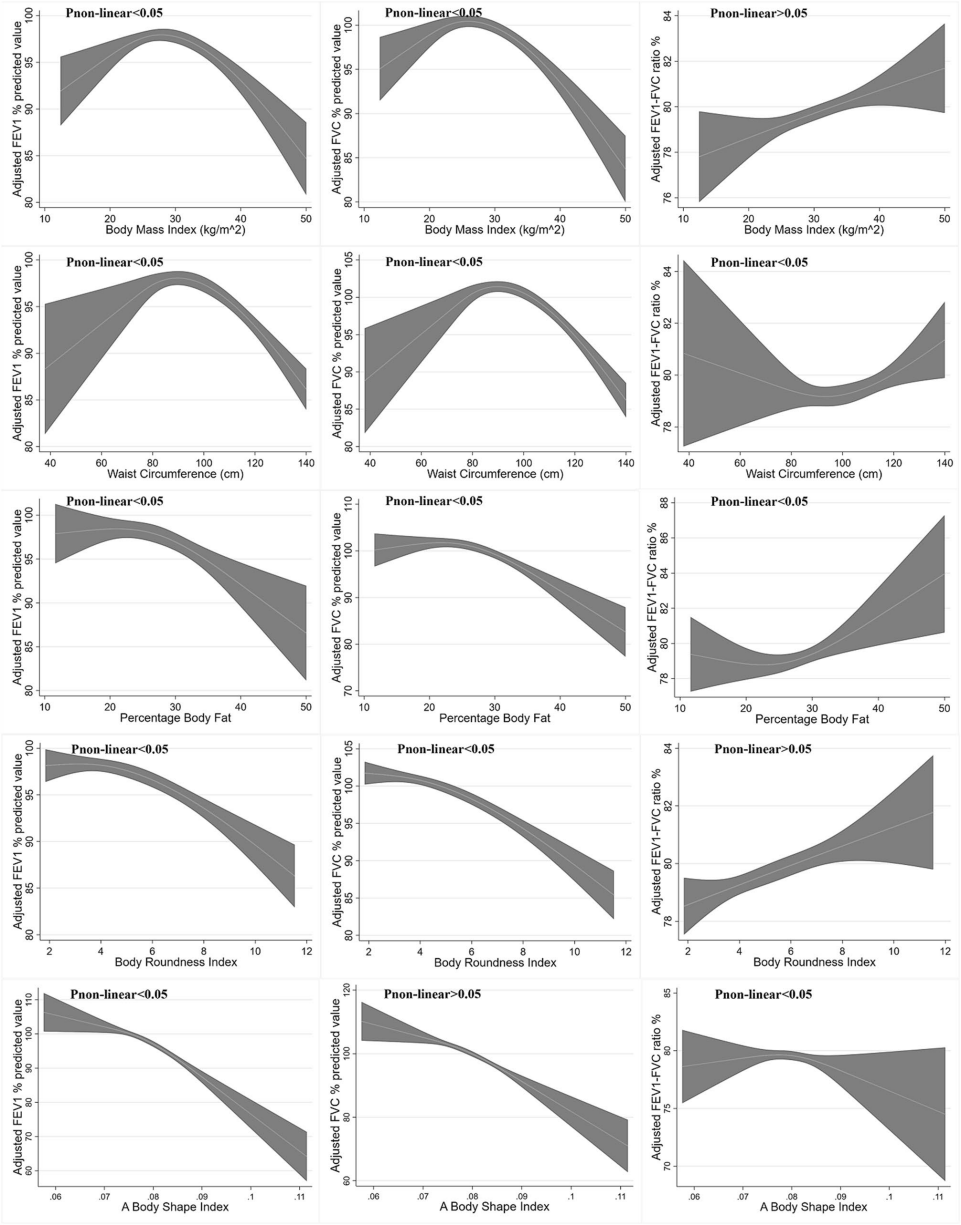
Reverse U-shaped association between BMI, WC, PBF, BRI, ABSI and pulmonary function in male. Data were weighted estimates. The shadow area represents a 95% confidence interval. Pi_non-linear_ was estimated by a two-line piecewise linear model. The model was adjusted for age, race, education, smoking, physical activity.

After adjusting for age, race, education, smoking, and physical activity, associations between BMI, WC, PBF, BRI, ABSI and FEV_1,_ as well as FVC were reverse U-shaped in both sexes (Figs. 2 and 3). The FEV_1_ and FVC reached a peak at BMIs in the overweight range, with positive associations below and inverse associations above the range. In this way, the decrease of FEV_1_ and FVC after BMI change point seemed to be more profound in males than in females. Additionally, BMI was positively associated with FEV_1_/FVC ratio, where nonlinearity only existed in the female participants. The similar shape of non-linear associations was also found when the relation between WC, PBF, BRI, ABSI and lung function values was evaluated. This non-linear associations still existed after transforming these anthropometric measurements into age and sex standardized Z-score (Supplementary Fig. 2).

We further validated the precise non-linear association and excluded the possibility of artificial nonlinearity. As exhibited in Table 3, Figs. 2 and 3, The non-linearity change points for pulmonary function were approximately within the second tertile of anthropometric measures. Thus, a segmented, multivariable linear regression was performed. The results validated the reversed U-shaped association between BMI and FEV_1_ and FVC in both female and male participants. Furthermore, these reversed U-shaped association was more profound in male participants with obesity, where the regression coefficient was almost twice as high as that in females (− 0.35 vs. − 0.19 for FEV_1_, − 0.42 vs. − 0.26 for FVC).

**Table 3:** Association between BMI, WC, PBF, BRI,ABSI and pulmonary function.

Female			
**BMI**	< 25.0	25.0–29.9	≥ 30
FEV_1_ %predicted	**0.80 [0.36, 1.24]**	−0.47 [−1.15, 0.20]	−0.19 [−0.39, 0.00]
FVC %predicted	**1.02 [0.62, 1.43]**	−0.61 [−1.23, 0.02]	**−0.26 [−0.44, −0.09]**
FEV_1_/FVC ratio %	−0.14 [−0.35, 0.07]	0.08 [−0.29, 0.45]	0.05 [−0.02, 0.13]
**Waist circumference (cm)**	Tertile 1 [< 86.6]	Tertile 2 [86.6–100.1]	Tertile 3 [> 100.1]
FEV_1_ %predicted	0.02 [−0.12, 0.16]	−0.17 [−0.35, 0.01]	**−0.13 [−0.22, −0.04]**
FVC %predicted	0.09 [−0.06, 0.23]	**−0.24 [−0.45, −0.04]**	**−0.17 [−0.24, −0.10]**
FEV_1_/FVC ratio %	−0.07 [–0.15, 0.00]	0.05 [−0.05, 0.15]	0.02 [−0.02, 0.06]
**Percentage body fat %**	Tertile 1 [< 37.2]	Tertile 2 [37.2–42.3]	Tertile 3 [> 42.3]
FEV_1_ %predicted	0.06 [–0.27, 0.39]	0.26 [−0.31, 0.84]	−0.31 [−0.82, 0.21]
FVC %predicted	0.22 [−0.06, 0.49]	−0.06 [–0.70, 0.56]	**−0.66 [−1.16, −0.15]**
FEV_1_/FVC ratio %	−0.12 [−0.27, 0.02]	0.27 [−0.06, 0.59]	**0.26 [0.05, 0.47]**
**Body roundness index**	Tertile 1 [< 4.04]	Tertile 2 [4.04–5.93]	Tertile 3 [> 5.93]
FEV_1_ %predicted	1.02 [−0.27, 2.30]	−1.14 [−2.53, 0.24]	−0.58 [−1.16, 0.01]
FVC %predicted	**1.71 [0.42, 3.01]**	−1.17 [−2.77, 0.43]	**−0.89 [−1.36, −0.42]**
FEV_1_/FVC ratio %	−0.51 [−1.02, 0.01]	0.02 [−0.94, 0.98]	0.22 [−0.03, 0.46]
**A body shape index (per 0.001 increment)**	Tertile 1 [< 0.0780]	Tertile 2 [0.0780–0.0819]	Tertile 3 [> 0.0819]
FEV_1_ %predicted	**−0.50 [−0.81, −0.20]**	−0.14 [−1.04, 0.76]	**−0.61 [−1.11, −0.11]**
FVC %predicted	**−0.58 [−0.88, −0.28]**	−0.37 [−1.24, 0.50]	**−0.44 [−0.75, −0.12]**
FEV_1_/FVC ratio %	0.02 [−0.14, 0.18]	0.10 [−0.24, 0.45]	−0.17 [−0.42, 0.07]
**Male**			
**BMI**	< 25.0	25.0–29.9	≥ 30
FEV_1_ %predicted	**1.29 [0.78, 1.81]**	**−0.68 [−1.27, −0.08]**	**−0.35 [−0.51, −0.19]**
FVC %predicted	**1.38 [0.93, 1.83]**	**−0.84 [−1.36, −0.31]**	**−0.42 [−0.56, −0.28]**
FEV_1_/FVC ratio %	−0.08 [−0.34, 0.17]	0.09 [−0.20, 0.37]	0.05 [−0.05, 0.15]
**Waist circumference (cm)**	Tertile 1 [< 92]	Tertile 2 [92–103.9]	Tertile 3 [> 103.9]
FEV_1_ %predicted	**0.22 [0.02, 0.42]**	−0.09 [−0.38, 0.20]	**−0.17 [−0.25, −0.10]**
FVC %predicted	**0.32 [0.14, 0.50]**	−0.10 [−0.32, 0.12]	**−0.20 [−0.27, −0.13]**
FEV_1_/FVC ratio %	**−0.10 [−0.18, −0.01]**	0.01 [−0.15, 0.17]	0.01 [−0.03, 0.06]
**Percentage body fat %**	Tertile 1 [< 25.3]	Tertile 2 [25.3–29.7]	Tertile 3 [> 29.7]
FEV_1_ %predicted	0.02 [−0.34, 0.37]	−0.35 [−1.23, 0.52]	−0.26 [−0.67, 0.15]
FVC %predicted	0.19 [−0.08, 0.45]	−0.57 [−1.41, 0.27]	−0.28 [−0.64, 0.09]
FEV_1_/FVC ratio %	−0.15 [−0.42, 0.12]	0.14 [−0.24, 0.53]	−0.02 [−0.16, 0.13]
**Body roundness index**	Tertile 1 [< 3.82]	Tertile 2 [3.82–5.25]	Tertile 3 [> 5.25]
FEV_1_ %predicted	1.43 [−0.09, 2.95]	**−2.83 [−4.88, −0.77]**	**−1.29 [−1.81, −0.78]**
FVC %predicted	**1.91 [0.53, 3.30]**	**−2.61 [−4.13, −1.09]**	**−1.41 [−1.93, −0.89]**
FEV_1_/FVC ratio %	−0.42 [−1.14, 0.30]	−0.15 [−1.26, 0.96]	0.06 [−0.20, 0.33]
**A body shape index (per 0.001 increment)**	Tertile 1 [< 0.0789]	Tertile 2 [0.0789–0.0825]	Tertile 3 [> 0.0825]
FEV_1_ %predicted	**−0.39 [−0.73, −0.06]**	**−1.26 [−2.30, −0.21]**	**−1.17 [−1.63, −0.70]**
FVC %predicted	−0.25 [−0.58, 0.08]	**−1.19 [−2.03, −0.36]**	**−0.99 [−1.43, −0.56]**
FEV_1_/FVC ratio %	−0.12 [−0.35, 0.11]	−0.01 [−0.49, 0.47]	−0.17 [−0.42, 0.08]

Data were weighted estimates and expressed as mean [95% confidence interval]. Multiple linear regression was adjusted for age, race, education level, physical activity (MET score), smoking status. For example, for female participants with BMI < 25.0 kg/m^2^, BMI was associated increased FEV_1_ %predicted value after adjusting for confounder above (coefficient = 0.80, 95% CI 0.36–1.24, P < 0.05).

Bold: p < 0.05.

## Discussion

In this study, we demonstrated the non-linear association between anthropometric measures (BMI, WC, PBF, BRI and ABSI) and pulmonary function. First, we confirmed in a national cohort that both underweight and obesity classifications according to BMI, were associated with reduced FEV_1_ and FVC. Moreover, after adjusting for age, sex, race, education, smoking, and physical activity, associations between BMI and FEV_1,_ as well as between BMI and FVC were reversed U-shaped. Finally, similar non-linear association shape was observed in WC, PBF, BRI and ABSI.

This nonlinear association has been indicated by a well-performed meta-analysis^11^, in which both normal weight and overweight had similar pulmonary functions regarding FEV_1_ and FVC. A simple linear model cannot well-explain this phenomenon. In our study, using restricted cubic spline, we initially demonstrated the non-linear association between BMI and pulmonary function. BMI does not distinguish between muscle and body fat. In addition to BMI, we also examined the anthropometrics which measures abdominal and visceral adiposity, including WC, PBF, BRI and ABSI. This suggested, adiposity was associated with impaired pulmonary function, only after certain thresholds. ABSI, to a lesser extent, corrected nonlinearly only with FEV_1_. These results may have potential influence on further studies. First, because the low pulmonary function is a risk factor for future all-cause mortality and cardiovascular events^12^. It is reasonable to assume that reduced pulmonary function partially mediated the deleterious effect of obesity on all-cause mortality and cardiovascular events. Indeed, previous studies have observed that BMI and other anthropometrics had a U-shaped or J-shaped association with overall mortality and cardiovascular events^13–15^. Second, BMI, WC, PBF, BRI, and ABSI should be treated as confounding factors with cautions in studies regarding pulmonary function. The unawareness of this nonlinear association between anthropometric measures and pulmonary function may bring potential bias.

Although the pathogenic mechanisms are not yet fully understood, plausible mechanisms may be proposed to explain the distinctive association between obesity and pulmonary function. The most direct mechanism involves the deleterious effect of adiposity on abdominal and intercostal muscle strength^16^. Obesity directly alters the mechanical properties of the lungs and chest wall through the accumulation of fat in the mediastinum and the abdominal and thoracic cavities^17^. This action elevates the diaphragm and also limits its downward excursion, causing pleural pressure to increase and functional residual capacity to decrease^18^. Furthermore, obesity has been reported to reduce mobility, neural adaptations, and changes in muscle morphology, resulting in poorer muscle quality than in participants with normal weight^16^.

A second potential mechanism for this pathophysiologic process involves insulin resistance, and is closely linked to excess body fat^18,19^. In this study, we observed that obesity was associated with lower FEV_1_, FVC and higher FEV_1_/FVC ratio only in the highest insulin resistance tertile (Supplementary Table 3). Previous cross-sectional data of 922 nondiabetic participants in the Normative Aging Study also found that fasting insulin and insulin resistance were negatively correlated with FVC and FEV_1_^20^. A similar result was also found in the Strong Heart Study among adult American Indians^21^. Furthermore, the association of insulin resistance and pulmonary function was more profound with FVC values^22^. In the British Women’s Heart and Health Study, for example, insulin resistance increased by 5% for a one standard deviation decrease in FVC, and 3% for FEV_1_, resulting in an elevated FEV_1_/FVC ratio^23^. Thus, insulin resistance may act as a key mediator in the association between obesity and pulmonary function.

Another potential mechanism for this pathophysiologic process involves low-grade chronic inflammation. Chronic inflammation has been found with obesity in the absence of overt infection^24^. Our study also revealed that participants with obesity had a significantly higher level of C-reactive protein. Moreover, like insulin resistance, obesity was associated with lower FEV_1_, FVC and higher FEV_1_/FVC ratio only in the highest CRP tertile. In cross-sectional studies, strong inverse associations were found between CRP levels and quartiles of FEV_1_ among 1,131 healthy participants^25^ and a population with metabolic syndrome and diabetes^26^. Similarly, data from the British Regional Heart Study demonstrated significant inverse associations of baseline FVC and FEV_1_ with blood markers of inflammation, including CRP^27^. Thus, low-grade chronic inflammation may also act as a key mediator in the association between obesity and pulmonary function.

However, this evidence cannot explain the observation that underweight participants had an even greater decreased pulmonary function than the participants with obesity (Table 2). In addition to the NHANES 2007–2012 population, this phenomenon has been observed in Korea National Health and Nutrition Examination Survey participants^10^, and in participants of the European Community Respiratory Health Survey and Swiss Cohort Study on Air Pollution and Lung and Heart Diseases in Adults^28,29^. Underweight participants have a significantly higher rate of restrictive pulmonary dysfunction, defined as FEV_1_/FVC ratio ≥ lower limit of normal (LLN) and FVC < LLN^28^. Therefore, different mechanisms may be involved in both extremes of BMI. Indeed, BMI does not distinguish between muscle and body fat, while other measurements reflect less muscle component. This can explain the finding that a positive association between BMI and pulmonary function under its change points, rather than among other anthropometric measurements. (Supplementary Fig. 2). Smoking is more prevalent among underweight participants^29^. We thus performed a sensitivity analysis, which excluded the possibility of residual confounding by smoking. In contrast to insulin resistance and inflammation in those with obesity, low muscle mass in underweight participants is one of the possible reasons for the impaired lung function^10^. In our study, BRI and ABSI were closer to linear association with pulmonary function. Compared to BRI, ABSI is independent from BMI. As exhibited by previous study, muscle strength was negatively correlated with ABSI. These results suggests that those with a more central body profile tend to be weaker in muscle strength than others with the same BMI^30^, resulting in to weaker forced expiratory volume and capacity. Because of our relatively small number of underweight participants, in-depth explorations are warranted in further studies.

Our study has many strengths, including the use of a national cohort, and it is the first to demonstrate a non-linear association of anthropometric measurements and pulmonary function. However, limitations should be mentioned. First, although our study mainly provided evidence that anthropometric measurements were negatively and non-linearly associated with pulmonary function, a causal association could not be demonstrated due to the cross-sectional nature. A further longitudinal study is needed to validate our results. Second, the association between obesity and pulmonary function no longer existed after additional adjustment for insulin resistance or CRP. We further found that obesity was associated with lower FEV_1_, FVC and higher FEV_1_/FVC ratio only in the highest insulin resistance/CRP tertile, indicating that both insulin resistance and low-grade chronic inflammation may lie on the association pathway between obesity and pulmonary function. Third, although we used a two-line piecewise linear model to estimate a single change point, the choice of change point may also be inaccurate. However, the inaccuracy may only slightly affect the regression coefficient, but not the non-linear conclusions. Fourth, compared to the excluded participants, the included individuals were younger, more likely to be male, more educated, and more physically active. BMI, WC, PBF BRI and ABSI were significantly lower in the included population (Supplementary Table 5). Thus, this selection procedure may potentially bias the results. Fifth, the inclusion and exclusion criteria of this study limited the generalizability of the results to the participants with diabetes and pulmonary comorbidities, adolescents, Asians, and other ethnic minorities. Moreover, it has been reported that variation across maneuvers within a single test is approximately 6%^31^, which may have led to unsupportable inferences about the statistical significance of observed differences. Lastly, residual, and unobserved confounding may potentially bias the results.

In conclusion, this cross-sectional study demonstrated that BMI, WC, PBF, BRI and ABSI are non-linearly associated with pulmonary function. Both underweight and obesity groups exhibited significantly reduced FEV_1_ and FVC measurements in comparison to normal-weight participants. Reduced pulmonary function is a risk factor for future all-cause mortality and cardiovascular events; thus, further longitudinal studies are needed to validate whether the nonlinearity can explain the U-shaped or J-shaped associations between BMI and the overall mortality and cardiovascular events.

## Methods

### Data source and participants

Data analyzed in this study were obtained from the 2007–2012 National Health and Nutrition Examination Survey (NHANES) data files because of the consistent pulmonary function test performed in those years. The NHANES, which is described in detail elsewhere, is a multistage probability sample of the noninstitutionalised U.S. population and allows representative estimates of the U.S. population. A total of 30,442 participants participated in NHANES from 2007 to 2012. We excluded participants who were: aged < 18 years (n = 11,823), ethnic minorities (n = 3612), without pulmonary function tests results (n = 3658), without pulmonary function tests that met American Thoracic Society (ATS) data collection standards (n = 1477), pregnant (n = 106), with pulmonary comorbidities, including asthma, chronic bronchitis, and emphysema (n = 1179), with diabetes (n = 1238), and without BMI information (n = 3). Diabetes was diagnosed based on self-report or the American Diabetes Association (ADA) standard (n = 1238)^32^. Ethnic minorities included races other than Mexican American, Non-Hispanic White, or Non-Hispanic Black, for they often lack corresponding spirometry reference values. Pulmonary comorbidities (including asthma, chronic bronchitis, and emphysema) were ascertained by self-report. In all, 7346 participants were included in this study. No participants underwent chest/abdominal surgery within three months prior to the lung function assessment. Comparison between included and excluded adults in this study is shown in Supplementary Table 1.

### Exposures and outcomes

The NHANES III Anthropometric Procedures Video (Centers for Disease Control and Prevention) illustrates the standard methodology for measuring body weight, height, WC, and skinfold thickness. The same methods were used in each of the continuous NHANES cycles. Based on the BMI, underweight, normal weight, overweight and obesity were diagnosed as: < 18·5 kg/m^2^; 18·5–24·9 kg/m^2^; 25·0–29·9 kg/m^2^; and ≥ 30·0 kg/m^2^, respectively^33^. The PBF was generated based on the age, race, height, weight, BMI, triceps skinfold thickness and subscapular skinfold thickness, waist and arm circumference^34^. Specifically, we applied Model G equation in our study^34^. This equation developed here is appropriate for use for multiple ethnic groups, is generalisable to the U.S. population, and provides a useful method for assessing the percent body fat. In addition to WC, we used BRI, a recently developed index of abdominal obesity^35^. The BRI was calculated as:

$${\text{BRI}} = 364.2 - 365.5 \times \sqrt {\frac{{\left( {\frac{WC}{{2\pi }}} \right)^{2} }}{{\left( {0.5 \times Height} \right)^{2} }}}$$.

We also used A Body Shape Index, an allometric transformed anthropometrics, to be another alternative approach to describe abdominal adiposity independent from BMI^36^. The ABSI was calculated: ABSI = WC × Weight^–2/3^ × Height^5/6^. It should be emphasised that we only generated PBF for participants in NHANES 2007–2010, as triceps or subscapular skinfold thickness were not measured in NHANES 2011–2012.

During NHANES 2007–2012, spirometry was offered to all the participants aged 6 to 79 years, except those with the following: current chest pain; a physical problem with forceful expiration; use of supplemental oxygen; recent surgery of the eye, chest, or abdomen; recent heart attack, stroke, tuberculosis exposure, or coughing up of blood; and history of detached retina, collapsed lung, or aneurysm. Similar spirometers (Ohio 822/827 dry-rolling seal volume spirometers, Ohio Medical, Gurnee, IL, USA) and protocols were used for conducting spirometry. Participants were asked to provide three acceptable maneuvers. For purposes of this study, we used pre-bronchodilator spirometry data with quality A (exceeds ATS data collection standards) or B (meets ATS data collection standards). The predicted values of pulmonary functions (FEV_1_, FVC) were calculated according to NHANES III equations^37^. In brief, age, sex, ethnicity groups, age^2^, and height^2^ were used to generate the predictive values.

### Covariables

The definitions and methods used for other baseline measurements (age, sex, ethnicity, weight, height, and WC) have been described in detail earlier. We defined “current smoker” as a participant who smoked every day. Insulin resistance was assessed by the homeostasis model of assessment for insulin resistance index (HOMA-IR), which was calculated as fasting plasma insulin (mU/L) × fasting plasma glucose (mmol/L)/22.5^38^. Physical activity was assessed by the metabolic equivalent of task (MET) score as follows: vigorous work-related activity × 8 + Moderate work-related activity × 4 + Walking or bicycling for transportation × 4 + Vigorous leisure-time physical activity × 8 + Moderate leisure-time physical activity × 4. Any missing MET score were regarded as 0. The MET expresses the energy cost of physical activities as a multiple of the resting metabolic rate.

### Statistical analysis

Analysis of variance (ANOVA) and Bonferroni method for multiple comparisons were used to determine differences in cross-sectional characteristics between groups, for continuous data with a normal and non-normal distribution, after the log-transformation. The chi-squared test was used for dichotomous and categorical data. Multiple linear regression models were applied to assess multivariable associations between body weight and pulmonary function parameters. Physical activity (MET score) was first transformed to categorical variable based on quartiles and then used in the regression analysis. Where there was evidence of non-linearity in Kernel-weighted local polynomial smoothing, a two-line piecewise linear model with a single change point was estimated by including all possible values for the change point and choosing the value with the highest likelihood. Then we applied a restricted cubic spline with three knots to explore the non-linear association. The first and last knots were placed at percentage 1 and percentage 99 of the examined parameters, and the middle one was placed at the point chosen by the two-line piecewise linear model. As obesity’s magnitude and distribution are dissimilar according to sex, results are shown for the males and females separately.

A 2-sided p-value < 0.05 was considered for statistical significance. All analyses were weighted to represent the U.S. population and to account for the intricate survey design and was performed in Stata version 15.0 (StataCorp LLC, College Station, TX, USA).

## Supplementary Information

Supplementary Information.

## Data Availability

The study was approved by the National Center for Health Statistics institutional review board, and written informed consent was received from all participants. All methods were carried out in accordance with relevant guidelines and regulations.
