# Supplementary material for: Non-linear association of anthropometric measurements and pulmonary function
Source: Sci Rep. 2021 Jul 16;11:14596. doi: 10.1038/s41598-021-93985-0 (PMC8285490; doi:10.1038/s41598-021-93985-0)
Supplement: Supplementary file 1 — Supplementary Information. [file 41598_2021_93985_MOESM1_ESM.docx]

**Supplementary Figure 1.** Scatter plot displaying the relationships between and anthropometric measurements


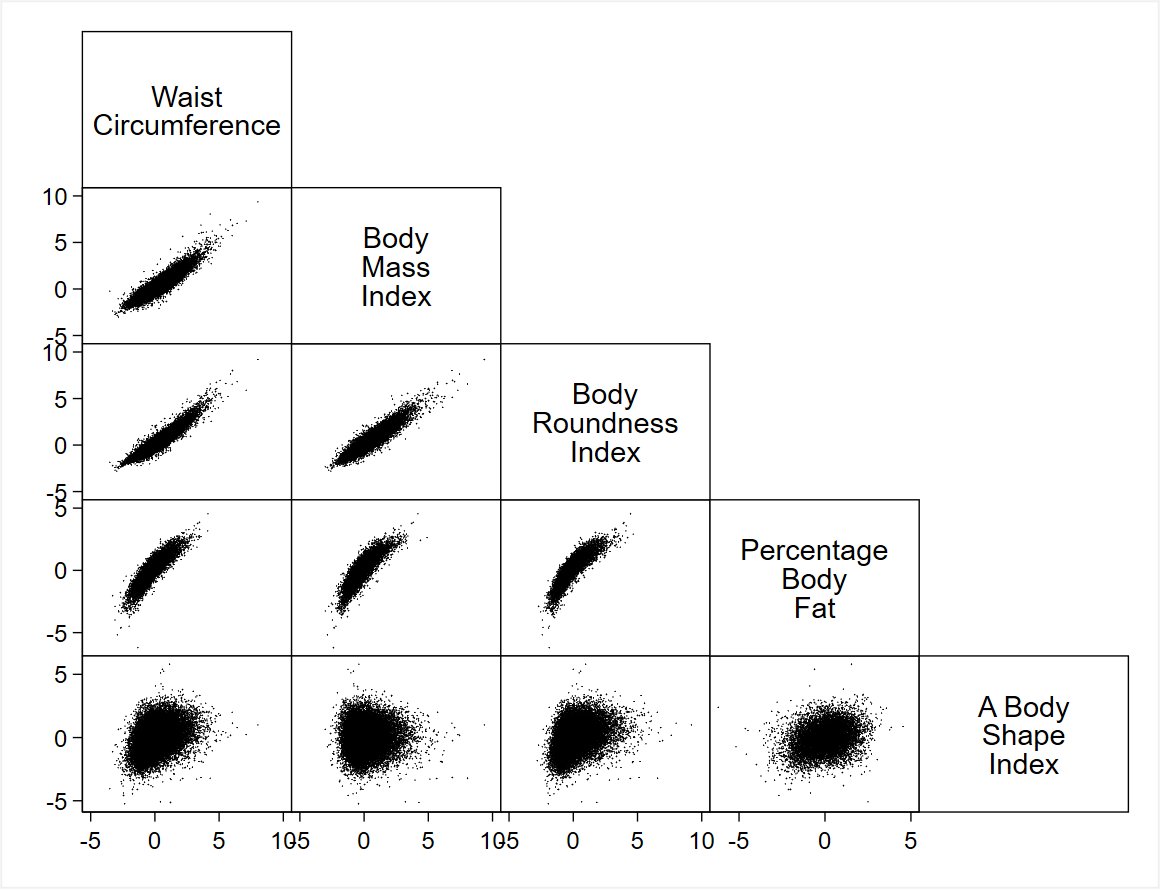


Data were transformed into age and sex standardized Z-score.

**Supplementary Figure 2.** Reverse U-shaped association between BMI, WC, PBF, BRI, ABSI (age and sex standardized Z-score) and pulmonary function


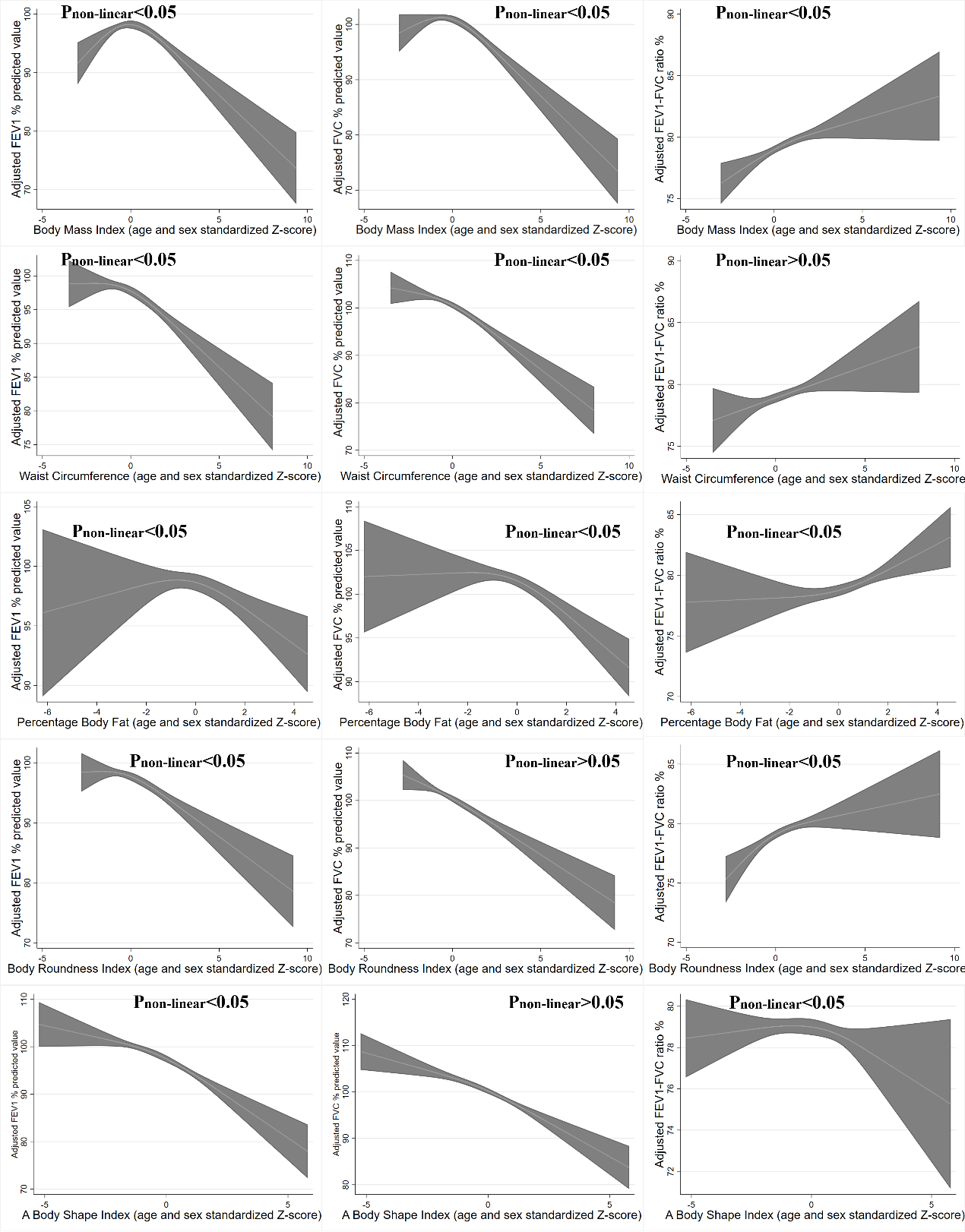


Data were weighted estimates. The shadow area represents a 95% confidence interval. Pi_non-linear_ was estimated by a two-line piecewise linear model. The model was adjusted for age, race, education, smoking, physical activity

**Supplementary Table 1.** the relationships between BMI (age and sex standardized Z-score) and anthropometric measurements (age and sex standardized Z-score)

|  | BMI (kg/m^2^) | | Waist circumference (cm) | | Percentage Body Fat (%) | | Body roundness index | |
| --- | --- | --- | --- | --- | --- | --- | --- | --- |
|  | Coefficient | R-squared | Coefficient | R-squared | Coefficient | R-squared | Coefficient | R-squared |
| BMI (kg/m^2^) |  | |  | |  | |  | |
| Waist circumference (cm) | 0.91 [0.90-0.93] | 0.864 |  | |  | |  | |
| Percentage Body Fat (%) | 0.70 [0.68-0.72] | 0.761 | 0.76 [0.74-0.77] | 0.788 |  | |  | |
| Body roundness index | 0.93 [0.92-0.94] | 0.883 | 0.96 [0.95-0.97] | 0.917 | 1.10 [1.07-1.13] | 0.779 |  | |
| A body shape index | 0.03 [-0.01,0.07] | 0.001 | 0.37 [0.343-0.40] | 0.127 | 0.27 [0.24-0.30] | 0.073 | 0.34 [0.31-0.37] | 0.101 |

Data were weighted estimates and expressed as mean [95% confidence interval].

**Supplementary Table 2.** Association of body weight and pulmonary function by sex

|  |  | Underweight | Normal weight | Overweight | Obesity |
| --- | --- | --- | --- | --- | --- |
|  |  | (n=108) | (n=2198) | (n=2557) | (n=2483) |
| Model 1 |  |  |  |  |  |
| Female | FEV1 %predicted | **-8.35 [-14.24, -2.47]** | Reference | 1.33 [-0.79, 3.45] | -0.56 [-3.49, 2.37] |
|  | FVC %predicted | **-9.64 [-11.17, -5.12]** | Reference | 0.72 [-0.92, 2.37] | -1.46 [-4.19, 1.28] |
|  | FEV1/FVC ratio % | 0.45 [-2.11, 3.01] | Reference | 0.34 [-0.67, 1.36] | ‘0.62 [-0.59, 1.83] |
| Male | FEV1 %predicted | **-10.11 [-19.21, -1.03]** | Reference | **2.66 [0.62, 4.69]** | **2.75 [0.11, 5.39]** |
|  | FVC %predicted | **-12.34 [-22.48, -2.19]** | Reference | 1.47 [-0.17, 3.12] | 1.33 [-1.04, 3.70] |
|  | FEV1/FVC ratio % | 1.84 [-7.58, 11.25] | Reference | 0.97 [-0.23, 2.17] | 1.23 [-0.39, 2.84] |
| Model 2 |  |  |  |  |  |
| Female | FEV1 %predicted | **-7.45 [-11.47, -3.44]** | Reference | 1.24 [-0.31, 2.80] | **2.20 [0.03, 4.38]** |
|  | FVC %predicted | **-9.31 [-12.62, -6.00]** | Reference | 0.75 [-0.74, 2.25] | 0.24 [-1.71, 2.19] |
|  | FEV1/FVC ratio % | 1.15 [-0.69, 2.99] | Reference | 0.33 [-0.55, 1.21] | **1.44 [0.71, 2.17]** |
| Male | FEV1 %predicted | **-6.99 [-13.26, -0.72]** | Reference | **1.86 [0.03, 3.69]** | 0.65 [-1.25, 2.55] |
|  | FVC %predicted | **-8.10 [-14.81, -1.40]** | Reference | 0.52 [-1.19, 2.22] | -1.48 [-3.32, 0.35] |
|  | FEV1/FVC ratio % | 1.32 [-4.01, 6.66] | Reference | **1.05 [0.02, 2.08]** | **1.58 [0.44, 2.72]** |
| Model 3 |  |  |  |  |  |
| Female | FEV1 %predicted | -8.13 [-16.88, 0.61] | Reference | **2.77 [0.78, 4.75]** | 3.26 [-0.31, 6.82] |
|  | FVC %predicted | **-10.16 [-16.54, -3.78]** | Reference | **2.17 [0.49, 3.85]** | 1.94 [-1.45, 5.33] |
|  | FEV1/FVC ratio % | 0.87 [-2.76, 4.50] | Reference | 0.32 [-0.95, 1.59] | 0.96 [-0.48, 2.40] |
| Male | FEV1 %predicted | -7.66 [-17.15, 1.82] | Reference | **2.70 [0.12, 5.29]** | 2.93 [-0.22, 6.09] |
|  | FVC %predicted | -9.8 [-21.02, 1.40] | Reference | 1.88 [-0.13, 3.89] | 1.96 [-1.21, 5.13] |
|  | FEV1/FVC ratio % | 2.11 [-8.30, 12.52] | Reference | 0.80 [-0.60, 2.19] | 1.15 [-1.03, 3.32] |

Data were weighted estimates and expressed as mean [95% confidence interval].

Multiple linear regression was adjusted for Model 1: age, race, education level, physical activity (MET score), smoking status and log-transformed insulin resistance; Model 2: age, race, education level, physical activity (MET score), smoking status and log-transformed C-reactive protein; Model 3: age, race, education level, physical activity (MET score), smoking status, log-transformed insulin resistance and log-transformed C-reactive protein

**Bold**: p<0.05;

| Insulin resistance | | Normal weight | Obesity with IR tertile 1 | Obesity with IR tertile 2 | Obesity with IR tertile 3 |
| --- | --- | --- | --- | --- | --- |
| Female |  |  | IR (0.48-2.12) n=187 | IR (2.82-4.67) n=187 | IR (4.68-22.52)  n=188 |
|  | FEV1 %predicted | Reference | 0.56 [-2.29, 3.41] | -2.52 [-5.57, 0.52] | **-3.72 [-6.46, -0.98]** |
|  | FVC %predicted | Reference | -0.31 [-3.21, 2.59] | **-4.72 [-7.12, -2.33]** | **-6.18 [-9.20, -3.15]** |
|  | FEV1/FVC ratio % | Reference | 0.74 [-0.51, 1.99] | **1.47 [0.16, 2.78]** | **1.71 [0.26, 3.05]** |
| Male |  |  | IR (0.70-3.09) n=157 | IR (3.10-5.15) n=157 | IR (5.17-24.42)  n=157 |
|  | FEV1 %predicted | Reference | 3.01 [-0.11, 6.14] | -1.17 [-3.52, 1.18] | **-3.36 [-6.17, -0.55]** |
|  | FVC %predicted | Reference | 1.65 [-1.07, 4.36] | **-2.68 [-5.23, -0.13]** | **-6.02 [-8.37, -3.67]** |
|  | FEV1/FVC ratio % | Reference | 1.19 [-0.45, 2.83] | 1.01 [-0.43, 2.44] | **1.85 [0.53, 3.17]** |
| C-reactive protein | | Normal weight | Obesity with CRP tertile 1 | Obesity with CRP tertile 2 | Obesity with CRP tertile 3 |
| Female |  |  | CRP (0.01-0.26) n=302 | CRP (0.27-0.65) n=295 | CRP (0.66-4.68) n=310 |
|  | FEV1 %predicted | Reference | 2.03 [-0.18, 4.23] | -1.50 [-3.91, 0.91] | **-3.09 [-4.77, -1.40]** |
|  | FVC %predicted | Reference | 0.12 [-1.87, 2.12] | **-2.35 [-4.14, -0.55]** | **-5.58 [-7.24, -3.92]** |
|  | FEV1/FVC ratio % | Reference | **1.36 [0.69, 2.04]** | 0.39 [-0.72, 1.49] | **1.64 [0.87, 2.41]** |
| Male |  |  | CRP (0.01-0.15) n=273 | CRP (0.16-0.36) n=271 | CRP (0.37-8.76) n=276 |
|  | FEV1 %predicted | Reference | 0.24 [-1.86, 2.34] | -1.43 [-3.66, 0.79] | **-3.68 [-5.63, -1.72]** |
|  | FVC %predicted | Reference | -0.41 [-2.57, 1.76] | **-3.59 [-5.53, -1.66]** | **-6.42 [-8.24, -4.61]** |
|  | FEV1/FVC ratio % | Reference | 0.41 [-0.86, 1.67] | **1.53 [0.44, 2.62]** | **2.02 [0.99, 3.05]** |

**Supplementary Table 3.** Association of obesity, insulin resistance and pulmonary function by sex

Data were weighted estimates and expressed as mean [95% confidence interval].

Multiple linear regression was adjusted for: age, race, education level, physical activity (MET score), smoking status.

**Bold**: p<0.05;

**Supplementary Table 4.** Association of body weight and pulmonary function among non-smokers

|  |  | Underweight | Normal weight | Overweight | Obesity |
| --- | --- | --- | --- | --- | --- |
| Female |  | (n=53) | (n=931) | (n=929) | (n=1129) |
|  | FEV1 %predicted | -4.20 [-8.77, 0.38] | Reference | 0.17 [-1.06, 1.41] | **-2.30 [-3.80, -0.79]** |
|  | FVC %predicted | **-7.22 [-11.17, -3.28]** | Reference | 0.05 [-1.22, 1.32] | **-3.34 [-4.70, -1.98]** |
|  | FEV1/FVC ratio % | **2.39 [0.78, 4.01]** | Reference | -0.08 [-0.78, 0.62] | 0.56 [-0.13, 1.26] |
| Male |  | (n=17) | (n=760) | (n=1174) | (n=991) |
|  | FEV1 %predicted | **-8.88 [-14.78, -2.99]** | Reference | 0.66 [-0.96, 2.28] | **-2.15 [-3.68, -0.61]** |
|  | FVC %predicted | **-9.13 [-14.51, -3.76]** | Reference | -0.10 [-1.56, 1.36] | **-3.26 [-4.71, -1.82]** |
|  | FEV1/FVC ratio % | 0.47 [-3.36, 4.31] | Reference | 0.55 [-0.23, 1.32] | 0.78 [-0.05, 1.62] |

**Supplementary Table 5.** Cross-sectional characteristics of included and excluded participants

| Characteristic | Excluded participants (n=11273) | Included participants (n=7346) | p value |
| --- | --- | --- | --- |
| Weighted proportion to all adults% | 50.8 (1.0) | 49.2 (1.0) |  |
| Age (n=18619) | 49.2 (0.4) | 43.1 (0.4) | <0.001 |
| Male (%) (n=18619) | 47.1 (0.5) | 49.6 (0.7) | 0.01 |
| Ethnicity % (n=18619) |  |  | NC |
| Mexican American | 7.0 (0.9) | 9.8 (1.2) |  |
| Non-Hispanic White | 56.2 (2.4) | 79.3 (1.8) |  |
| Non-Hispanic Black | 12.1 (1.1) | 11.0 (1.1) |  |
| Education level (%) (n=18571) | 23.6 (1.1) | 30.7 (1.4) | <0.001 |
| Current smoker % (n=18619) | 16.4 (0.6) | 17.5 (0.9) | 0.21 |
| MET scores /week (n=18619) | 240 [0-960] | 480 [120-1440] | <0.001 |
| HOMA-IR (n=7477) | 2.8 [1.6-5.0] | 2.2 [1.4-3.6] | <0.001 |
| C-Reactive Protein (mg/dL) (n=11598) | 0.19 [0.07-0.46] | 0.15 [0.06-0.35] | <0.001 |
| BMI (kg/m2) (n=17695) | 29.1 (0.1) | 28.1 (0.1) | <0.001 |
| Height (cm) (n=17724) | 167.4 (0.2) | 170.1 (0.1) | <0.001 |
| Waist circumference (cm) (n=16896) | 99.5 (0.4) | 96.3 (0.3) | <0.001 |
| Percentage Body Fat (%)(n=9713) | 34.2 (0.2) | 33.0 (0.2) | <0.001 |
| Body roundness index (n=16874) | 5.52 (0.06) | 4.86 (0.04) | <0.001 |
| A Body Shape Index (n=16856) | 0.0819 (0.0001) | 0.0804 (0.0001) | <0.001 |
| FEV_1_ (%predicted) (n=11349) | 91.5 (0.4) | 97.7 (0.3) | NC |
| FVC (%predicted) (n=11349) | 95.1 (0.4) | 100.4 (0.3) | NC |
| FEV_1_/FVC ratio (n=14194) | 78.1 (0.3) | 78.4 (0.2) | NC |

The excluded participants were defined as those with age≥18 but did not meet other inclusion criteria. Data were weighted estimates and expressed as mean (standard error) or median [percentile 25 -percentile 75] (n= number of participants in analysis). Education level, percentage of participants, completed college graduate or above; HOMA-IR, homeostasis model of assessment for insulin resistance index. MET score, metabolic equivalent scores per week

*p<0.05 compared to normal weight; NC: not comparable.
